# Outcomes in Trials for Management of Caries Lesions (OuTMaC): protocol

**DOI:** 10.1186/s13063-015-0927-3

**Published:** 2015-09-07

**Authors:** Falk Schwendicke, Thomas Lamont, Nicola Innes

**Affiliations:** Department of Operative and Preventive Dentistry, Charité – Universitätsmedizin, Aßmannshauser Str. 4-6, 14197 Berlin, Germany; Dundee Dental School, University of Dundee, Park Place, Dundee, Tayside UK

**Keywords:** Dental caries, Clinical trial, Delphi, Dental, Outcomes, Review

## Abstract

**Background:**

Clinical trials on caries lesion management use an abundance of outcomes, hampering comparison or combination of different study results and their efficient translation into clinical practice. Core outcome sets are an agreed standardized collection of outcomes which should be measured and reported in all trials for a specific clinical area. We aim to develop a core outcome set for trials investigating management of caries lesions in primary or permanent teeth conducted in primary or secondary care encompassing all stages of disease.

**Methods:**

To identify existing outcomes, trials on prevention and trials on management of caries lesions will be screened systematically in four databases. Screening, extraction and deduplication will be performed by two researchers until consensus is reached. The definition of the core outcome set will by based on an e-Delhi consensus process involving key stakeholders namely patients, dentists, clinical researchers, health economists, statisticians, policy-makers and industry representatives. For the first stage of the Delphi process, a patient panel and a separate panel consisting of researchers, clinicians, teachers, industry affiliated researchers, policy-makers, and other interested parties will be held. An inclusive approach will be taken to involve panelists from a wide variety of socio-economic and geographic backgrounds. Results from the first round will be summarized and fed back to individuals for the second round, where panels will be combined and allowed to modify their scoring in light of the full panel’s opinion. Necessity for a third round will be dependent on the outcome of the first two. Agreement will be measured via defined consensus rules; up to a maximum of seven outcomes. If resources allow, we will investigate features that influence decision making for different groups.

**Discussion:**

By using an explicit, transparent and inclusive multi-step consensus process, the planned core outcome set should be justifiable, relevant and comprehensive. The dissemination and application of this core outcome set should improve clinical trials on managing caries lesions and allow comparison, synthesis and implementation of scientific data.

**Trial registration:**

Registered 12 April 2015 at COMET (http://www.comet-initiative.org)

## Background

Dental caries is one of the most commonly occurring diseases worldwide [1]. It has significant impact on patients resulting from pain, infection, and loss of functionality, and carries high lifelong costs from treating the disease and its symptoms when prevention has failed. Traditional curative dental care is the fourth most costly disease to treat in most industrialized countries [2]. Caries prevention has traditionally focused on non-operative measures (plaque and diet control, fluoride application), whilst caries treatment applies operative procedures, traditionally involving removal of caries before placing a restoration. However, recently, our understanding of the disease caries, as a biofilm disorder, has evolved. This understanding has led to novel non-operative and operative means for managing the disease or its symptoms. However, there remains uncertainty as to which caries management strategy is most suitable and under which circumstances, leading to wasteful use of healthcare resources and less-than-optimal outcomes. Part of this uncertainty stems from the abundance of outcomes being used in clinical trials, hampering comparison or combination of different study results [3, 4]. Consequently, trials on caries management are not efficient and their data are not being maximized. Information on treatments’ effectiveness and efficacy are not well communicated throughout the dental world, reducing the likelihood that the highest standard of care is being delivered.

The COMET (Core Outcome Measures in Effectiveness Trials) Initiative (http://www.comet-initiative.org) provides a forum to bring together researchers interested in the development, application and promotion of core outcome sets (COS) that are defined as an agreed standardized collection of outcomes which should be measured and reported, as a minimum, in all trials for a specific clinical area. Defining such COS involves systematically collecting existing outcomes, identifying other outcomes that may not have been investigated, categorizing them, ordering them according to agreed needs and relevance, and eventually deciding on a COS in consensus. This consensus process should ideally involve patients, clinicians, researchers, teachers and any other stakeholders [5]. Moreover, a COS should aim at being as inclusive, i.e. applicable in different countries and settings [6]. Developing a COS for management of dental caries would encourage trialists to use outcomes chosen by consensus that are relevant to patients and clinicians and can be compared with similar studies.

## Objectives

Our aim is to develop a COS for the management of existing carious lesions in both primary and permanent teeth using consensus across specialists in the area, patients and the public. The outcome set will be applicable to studies investigating management of caries lesions in primary or permanent teeth conducted in primary or secondary care encompassing all stages of disease. It will not be limited by health status, age or geographical location of trials. The COS will be developed for management of caries lesions including traditional preventive measures such as fluoride varnish and fissure sealants when they are used in the management of existing lesions. The primary goal of the interventions should be to treat the lesion, even where the secondary goal involves managing the pulp. The COS will not be applicable to treatments aimed directly at managing the dental pulp, although other groups may develop core outcomes for these fields and there may be overlap in the sets.

## Methods/Design

### Identifying existing knowledge

To identify all outcomes reported by randomized controlled studies on prevention and treatment of caries lesions in primary and permanent teeth, two search strategies will be developed; one for screening trials on prevention of caries lesion development, the other for management of caries lesions. Inclusion criteria will be systematic reviews of RCTs, or RCTs, on preventing or managing caries lesions. The reviews and trials will either:

- compare an intervention for preventing or managing the lesions to:
  - another prevention or management intervention or
  - no treatment; or
- compare interventions to support patients undergoing dental procedures related to caries

No restrictions with regards to setting or location and type of the prevented or treated lesion will be made.

Four electronic databases (Cochrane Central Register of Controlled Trials, Cochrane Database of Systematic Reviews, Medline via PubMed, Embase) will be searched using defined strategies, and de-duplicated to provide a single set of titles/abstracts. Titles and abstracts will be screened by two calibrated reviewers for eligibility, and consensus obtained by discussion or consulting a third reviewer. Data from eligible studies will be independently extracted by two reviewers using electronic spreadsheets until both parties agree that saturation has been reached and no new outcomes are being found. A list of outcomes will be compiled, and outcomes with different verbatim terms but similar meaning combined in a single term.

### Consensus process

The COS will be developed by a consensus process involving key stakeholders: namely, patients and members of the public, dentists, clinical researchers, health economists, statisticians, policy-makers and industry representatives. Panelists will give their informed consent prior to taking part in the consensus process. To increase uptake of the resulting COS, we will engage with the Cochrane Oral Health Group, clinical guideline developers, research funders, journal editors, regulators such as research ethics committees, and trial registries. We will attempt to ensure adequate representation from each of the stakeholder groups to improve the acceptance and implementation of the COS. No ethical approval will be required for this study according to the East of Scotland Research Ethics Service (reference 15/GA/0033).

We will attempt to gain consensus via a three-stage Delphi process using an online platform to support a specially designed e-Delphi portal. This has been chosen as the most inclusive and pragmatic method to include key stakeholders from different settings, backgrounds and geographical locations. If consensus cannot be reached on a full outcome set we will propose a smaller core set based on those outcomes that have agreement.

For the first stage of the Delphi process, there will be two panels created, a patient panel and a separate group consisting of researchers, clinicians, teachers, industry affiliated researchers, policy-makers, and other interested parties that are identified. The first round of the Delphi process will be held separately for each of the two panels and they will be combined in the later Delphi stages. Recruitment of patient panelists and members of the public will be performed within dental clinics (patients will be recruited from an existing pool in different clinics) in different countries. An inclusive approach will be taken aiming to involve patients from various socio-economic and geographic backgrounds. An invitation (plain language version sent where appropriate) with the background of the COS development process will be sent or given to potential panelists, providing them with additional information regarding the practicalities of the consensus process. Participants will be asked to reply to the invitation using the e-Delphi portal, and, to minimize attrition, only those accepting the invitation and confirming active participation will be included in the process. Panelists will be asked to review the list of outcomes and add any that they consider relevant but missing from the list. Panelists will be informed that a minimum of two participants will have to propose an outcome for it to be included in the next stage of the process. The resulting list will again be harmonized using this rule and with single terms being used for items mentioned with multiple names. For this complete list, panelists will then be asked to score each outcome using the scale proposed by the GRADE group (http://www.gradeworkinggroup.org), in which 1 to 3 signifies an outcome of limited importance, 4 to 6 important but not critical, and 7 to 9 critical.

The results from the two panels will be summarized and fed back to individuals for the second round. In the second round, the panels will be combined to give a single panel and members will be reminded of their first choice, and will be allowed to change their score in light of the panel’s overall opinion. If there is a risk in the second round of the patient voice not coming through we will consider possible management options such as weighting. As people who do not agree with the group may be less inclined to continue the process, reminders will be sent to participants who do not respond. This should help to minimize overestimation of the extent of the consensus.

Consensus that an outcome should be included in the COS, will be defined as 70 % or more of the respondents scoring it 7 to 9 and fewer than 15 % scoring it as 1 to 3 (e.g. outcome will be retained). Consensus that an outcome should not be included in the COS will be defined as 70 % or more scoring it as 1 to 3 and fewer than 15 % scoring it as 7 to 9 (outcome will be discarded). If we reach consensus on more than two and up to five items, we will stop the consensus process and these items will form the COS. If this is not achieved, the Delphi process will be re-run.

### Dissemination

**Fig. 1:**
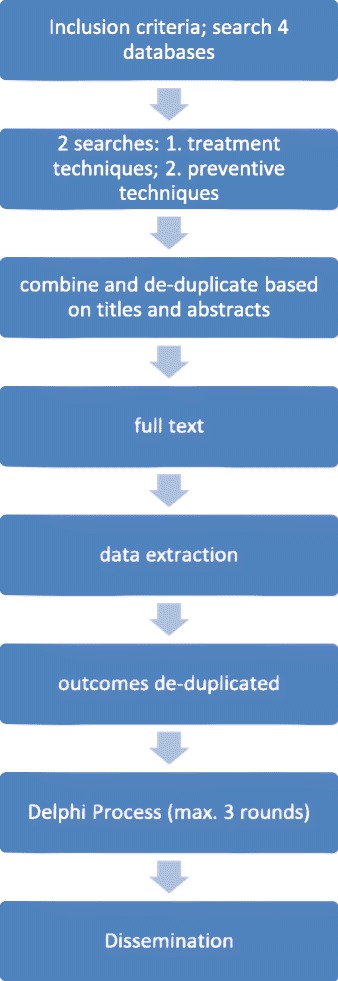
Study flow

To help increase the uptake of COS we will engage with relevant groups such as the Cochrane Oral Health Group, ORCA (the European Organization for Caries Research), the International Association for Dental Research Cariology Group, guideline development groups including National Institute for Clinical Excellence, the Scottish Intercollegiate Guideline Network and the Scottish Dental Clinical Effectiveness Program, journal editors, trial registries and major funding bodies such as the UK National Institute for Health Research (NIHR) Health Technology Assessment or the German Research Foundation. An overview of the trial can be found in Fig. 1.

## Discussion

Both current research in dentistry (or more specifically cariology) and its translation into clinical practice suffer from the lack of agreed, defined, standardized and comparable trial outcomes. For example, the Cochrane Oral Health Group database for reviews of “Dental caries treatment,” contains 12 reviews. Eight of these reported on less than two trials, whilst those four reviews that reported on two or more trials all commented on the inadequacy of applied outcome measurements (“lack of consistency” [7]; “insufficient outcome data on cost” [8]; “future research should investigate patient-centred outcomes …. Health economic measures should be used to determine the cost of treatment and patient willingness to pay” [3]). Thus, there is a need to agree on a defined set of minimum outcomes to be investigated and reported in dental clinical trials. Increasing the comparability between trials will allow more effective synthesis of data, which in turn will strengthen the overall evidence base and improve knowledge dissemination into clinical practice.

Besides allowing comparison between, and synthesis of, trials, defining and applying core outcomes might eventually also improve dental and caries research, as trialists will base their study evaluation on an agreed set of outcomes and (in a second step) outcome measures. Thus, future trial outcomes and results should be more comprehensive and relevant to all stakeholders in the field. To enhance this, we have built in broad stakeholder and patient and public involvement in the process of defining this COS. Using an agreed set of outcomes might, therefore, lead to future trial designs being more rigorous, transparent and accountable.

As described, involving stakeholders in the definition of the COS on caries lesion management trials is planned and desirable. So far, only one COS has been defined in dentistry using formal consensus methodology. This was for pulp therapy in primary teeth, and did not include patients in the process [9]. One of the factors likely to complicate the development of a COS for clinical trials of interventions for dental caries will be the non-existence of patient groups for dental caries (in contrast to other diseases or conditions such as cancer or cleft lip/palate). Thus, there is no option to approach a patient group during the definition of the panel members. We plan to invite patients from different clinical areas in dentistry; moreover, the use of e-Delphi should also lower the barriers for public and patient participation. From both a patient and a clinician perspective, management of dental caries has a number of situational-related influencing factors: for example, payment systems, priorities (aesthetics versus function) and differences placed on importance of the dentition across countries. The inclusion of the public, patients, researchers and other stakeholders from different countries will facilitate inclusion of groups from diverse backgrounds to help include these variety factors.

Finally, some might find a COS for clinical trials to be too rigid, restricting creative research. However, it will be important to emphasize for those unfamiliar with COS that the core outcomes are a *minimum* standard of outcomes to be assessed and reported. Researchers will be able to add other, specifically tailored outcomes to their planned trial, or assess outcomes which are found important after consulting patients or the wider public. In this sense, this COS will need adaption and regular updating as COS aim to reflect current agreed standards on trial objectives and priorities.

In conclusion, there is great need for a COS for trials on managing caries lesions. The planned development of such a set will involve a range of stakeholders, especially the public and patients. By using an explicit, transparent multi-step consensus process, this COS will be justifiable, relevant and comprehensive. The dissemination and application of this COS should improve clinical trials on managing caries lesions and allow comparison, synthesis and implementation of scientific data.

## Trial status

The review stage of this trial is initiated, i.e. search strategies have been developed and are currently being pilot-tested.

## Abbreviations

COMET: Core outcome measures in effectiveness trials
COS: Core outcome set
NIHR: National Institute for Health Research
ORCA: European Organization for Caries Research
RCT: Randomized controlled trial
